# Proteomic profile of mesothelial exosomes isolated from peritoneal dialysis effluent of children with focal segmental glomerulosclerosis

**DOI:** 10.1038/s41598-021-00324-4

**Published:** 2021-10-21

**Authors:** Maurizio Bruschi, Edoardo La Porta, Isabella Panfoli, Giovanni Candiano, Andrea Petretto, Enrico Vidal, Xhuliana Kajana, Martina Bartolucci, Simona Granata, Gian Marco Ghiggeri, Gianluigi Zaza, Enrico Verrina

**Affiliations:** 1 Laboratory of Molecular Nephrology, IRCCS Istituto Giannina Gaslini, Via Gerolamo Gaslini, 5, 16147 Genoa, Italy; 2 Dialysis Unit, Department of Pediatric, IRCCS Istituto Giannina Gaslini, Genoa, Italy; 3 Department of Internal Medicine (DiMi), University of Genoa, Genoa, Italy; 4 Dipartimento di Farmacia (DIFAR), Università di Genova, Genoa, Italy; 5 Core Facilities - Clinical Proteomics and Metabolomics, IRCCS Istituto Giannina Gaslini, Genoa, Italy; 6 Department of Medicine DAME-Division of Pediatrics, University of Udine, Udine, Italy; 7 Renal Unit, Department of Medicine, University Hospital of Verona, Verona, Italy; 8 UO of Nephrology, Dialysis and Transplantation, IRCCS Istituto Giannina Gaslini, Genoa, Italy

**Keywords:** Biomarkers, Nephrology, Proteomics

## Abstract

Peritoneal dialysis (PD) is the worldwide recognized preferred dialysis treatment for children affected by end-stage kidney disease (ESKD). However, due to the unphysiological composition of PD fluids, the peritoneal membrane (PM) of these patients may undergo structural and functional alterations, which may cause fibrosis. Several factors may accelerate this process and primary kidney disease may have a causative role. In particular, patients affected by steroid resistant primary focal segmental glomerulosclerosis, a rare glomerular disease leading to nephrotic syndrome and ESKD, seem more prone to develop peritoneal fibrosis. The mechanism causing this predisposition is still unrecognized. To better define this condition, we carried out, for the first time, a new comprehensive comparative proteomic mass spectrometry analysis of mesothelial exosomes from peritoneal dialysis effluent (PDE) of 6 pediatric patients with focal segmental glomerular sclerosis (FSGS) *versus* 6 patients affected by other primary renal diseases (No FSGS). Our omic study demonstrated that, despite the high overlap in the protein milieu between the two study groups, machine learning allowed to identify a core list of 40 proteins, with ANXA13 as most promising potential biomarker, to distinguish, in our patient population, peritoneal dialysis effluent exosomes of FSGS from No FSGS patients (with 100% accuracy). Additionally, the Weight Gene Co-expression Network Analysis algorithm identified 17 proteins, with PTP4A1 as the most statistically significant biomarker associated to PD vintage and decreased PM function. Altogether, our data suggest that mesothelial cells of FSGS patients are more prone to activate a pro-fibrotic machinery. The role of the proposed biomarkers in the PM pathology deserves further investigation. Our results need further investigations in a larger population to corroborate these findings and investigate a possible increased risk of PM loss of function or development of encapsulating peritoneal sclerosis in FSGS patients, thus to eventually carry out changes in PD treatment and management or implement new solutions.

## Introduction

Peritoneal dialysis (PD), given its almost universal applicability, cost-effectiveness and the possibility of a home-based treatment, is the dialysis modality of choice for children affected by end stage kidney disease (ESKD) while awaiting for kidney transplantation^1^.

Despite the great number of studies in the field, limited progress has been made in PD treatment, mainly consisting in the optimization of composition and biocompatibility of dialysis fluids, and improvements in the management of dialysis-related local and systemic complication. Indeed, local and systemic toxicity in PD is still a major issue, and PD patients present a 40-fold higher mortality risk compared to the healthy population^2^. This renal replacement therapy (RRT) takes advantage of the semipermeable characteristics of the peritoneal membrane (PM) to manage solute removal and ultrafiltration with a combination of short and long intraperitoneal dwells of a patient tailored volume of dialysis solutions containing different concentrations of an osmotic agent (glucose mainly, but also icodextrin and amino acids)^1^.

PM is composed of a single layer of mesothelial cells with its associated extracellular matrix (ECM), containing capillaries and lymphatic vessels. PM allows transport and movement of fluids and substances across the peritoneal cavity^3^.

Depuration and ultrafiltration occur efficiently when a vital and well-structured PM is exposed to the hyperosmotic-hyperglycemic PD fluid. However long-term exposure to these solutions, together with predisposing genetic factors, may lead to chronic inflammation and oxidative stress that cause detachment of mesothelial cells, angiogenesis^3^ and the onset of tissue fibrosis with consequent PM failure and the need of PD treatment discontinuation.

When normal repair mechanisms break down, mesothelial cells take on a profibrotic role, secreting inflammatory and profibrotic mediators, differentiating and migrating into the injured tissues where they contribute to fibrogenesis. In addition to secreting proinflammatory mediators and contributing to both coagulation and fibrinolysis, mesothelial cells undergo epithelial-to-mesenchymal transition (EMT) and become fibrogenic cells^4,5^.

In rare cases, the prolonged inflammatory stimulus may induce the development of encapsulating peritoneal sclerosis (EPS), a rare life-threatening condition whose prevalence ranges from 1 to 7%^6^, depending on the reports, or higher in patients with long dialysis vintage^7^, also after kidney transplantation or switching to hemodialysis^8^. In children its prevalence is around 2%^9^. Although EPS is strongly associated with the duration of PD, the pathogenesis remains only partially understood.

However, some authors have suggested that patients with immuno-mediated kidney diseases, such as focal segmental glomerulosclerosis (FSGS), the most common cause of steroid resistant nephrotic syndrome in children may be more prone to develop rapid peritoneal fibrosis and EPS^10,11^. FSGS is characterized by an increase in the mesangial matrix in some glomeruli, with obliteration of capillary lumens, sclerosis, hyalinosis, foam cells, and adhesions to the Bowman’s capsule^10,11^.

Many reasons may explain this condition, but recent data suggest that the use of calcineurin inhibitors (CNI), such as tacrolimus and cyclosporine A, primary immunosuppressive agents employed in FSGS, and in general for corticosteroid-dependent and resistant nephrotic syndrome, seems to play a pivotal role^12,13^. CNI, in fact, may cause fibrosis in kidney and other organs through the increment of TGFβ1 expression and EMT activation^14,15^. TGFβ1 plays a central role in fibrosis and EMT also in PD patients^16,17^. Increasing evidence from a number of studies suggest to reduce the use of CNIs, due to their nephrotoxicity^18,19^. We believe that new insights on CNIs toxicity/ pro-fibrotic role in PD could contribute to increase the shift toward different therapeutic approaches, also as first line therapy. Additionally, inconsistent results have proposed an additional pro-fibrotic role of β-blocker administration (mainly atenolol and carvedilol), frequently used to treat hypertension in children with FSGS-induced nephrotic syndrome^20–22^.

Furthermore, the activation of TGF-beta pathway could be the common mechanism in FSGS and EPS^23–26^. A consistent number of EPS cases have been described in children with FSGS^9,27^. In a previous study analyzing the potential of PD patients with FSGS to develop fibrosis compared to No FSGS, we found that 36% of children affected by EPS displayed FSGS as primary kidney disease^12^. At the moment, the above-mentioned study represent the first clinical evidence, thus we investigated the biological machinery linking these two conditions in order to proceed more focused in further clinical investigations .

Exosomes are nano-sized extracellular vesicles originating from the endosomal pathway that exist in almost all body fluids and are promising source of biomarkers^28^. Exosomes carry a cargo of proteins, lipids and nucleic acids, that can participate in cell-to-cell signaling affecting nearby and distant cells^29^, playing a role in EMT and peritoneal fibrosis^30,31^, and in angiogenesis processes^32^.

In this study we aimed to investigate whether FSGS pediatric patients undergoing PD are more prone to develop PM fibrosis compared to no FSGS patients evaluating the protein content of mesothelial exosomes isolated from peritoneal dialysis effluent (PDE) by comparative proteomic analysis. Mesothelial exosomes were isolated and purified from PD pediatric patients with FSGS as primary renal disease (PRD) and from PD patients affected by other PRDs (No FSGS).

## Results

### Characterization of exosomes

Size of exosomes was confirmed by dynamic light scattering (DLS), revealing a Gaussian distribution profile with a typical mean peak at 100 ± 5 nm (Supplemental Figure S1A). Western blot analysis revealed that the exosomes of both groups were positive for mesothelin (MSLN), CD81 and CD63 but not for CD45 (Supplemental Figure S1B). Moreover, both groups of exosomes reveled also the negativity of principal CD of infiltrating cells of immune system such as CD3, CD4, CD8, CD68 and CD79a^33^ (Supplemental Figure S1B) showing a typical immunophenotype of exosomes derived from mesothelial cells. There was no difference in size or immunophenotype profile between exosomes isolated from FSGS or No FSGS patients. Same immunephenotype results were confirmed by the analysis of mass spectrometry data.

### Protein composition of exosomes

The protein composition of mesothelial exosomes from PDE of FSGS and No FSGS samples was determined by mass spectrometry. We identified 3612 proteins, 1122 (45%) of which were present in both samples. Besides, only 174 (7%) and 1194 (48%) proteins were exclusively found in the FSGS and No FSGS patients, respectively (Supplemental Figure S2A).

Despite considerable overlapping of protein identity between the two clinical groups, multidimensional scaling analysis evidenced a clear discrimination of the two conditions (Supplemental Figure S2B).

A co-expression network was constructed using the weighted gene co-expression network analysis (WGCNA). WGCNA clusters proteins into modules of co-expression profile, considered to be in a functional relationship with each other^20^.

We used WGCNA to identify which module and protein expression profile was functionally associated with dialysis vintage, peritoneal equilibration test of glucose dialysate to initial dialysate concentration ratio (PET D/D0 glucose) and with peritoneal equilibration test of creatinine dialysate-to-plasma concentration ratio (PET D/P creatinine) at PET, and FSGS or No FSGS samples.

**Figure 1:**
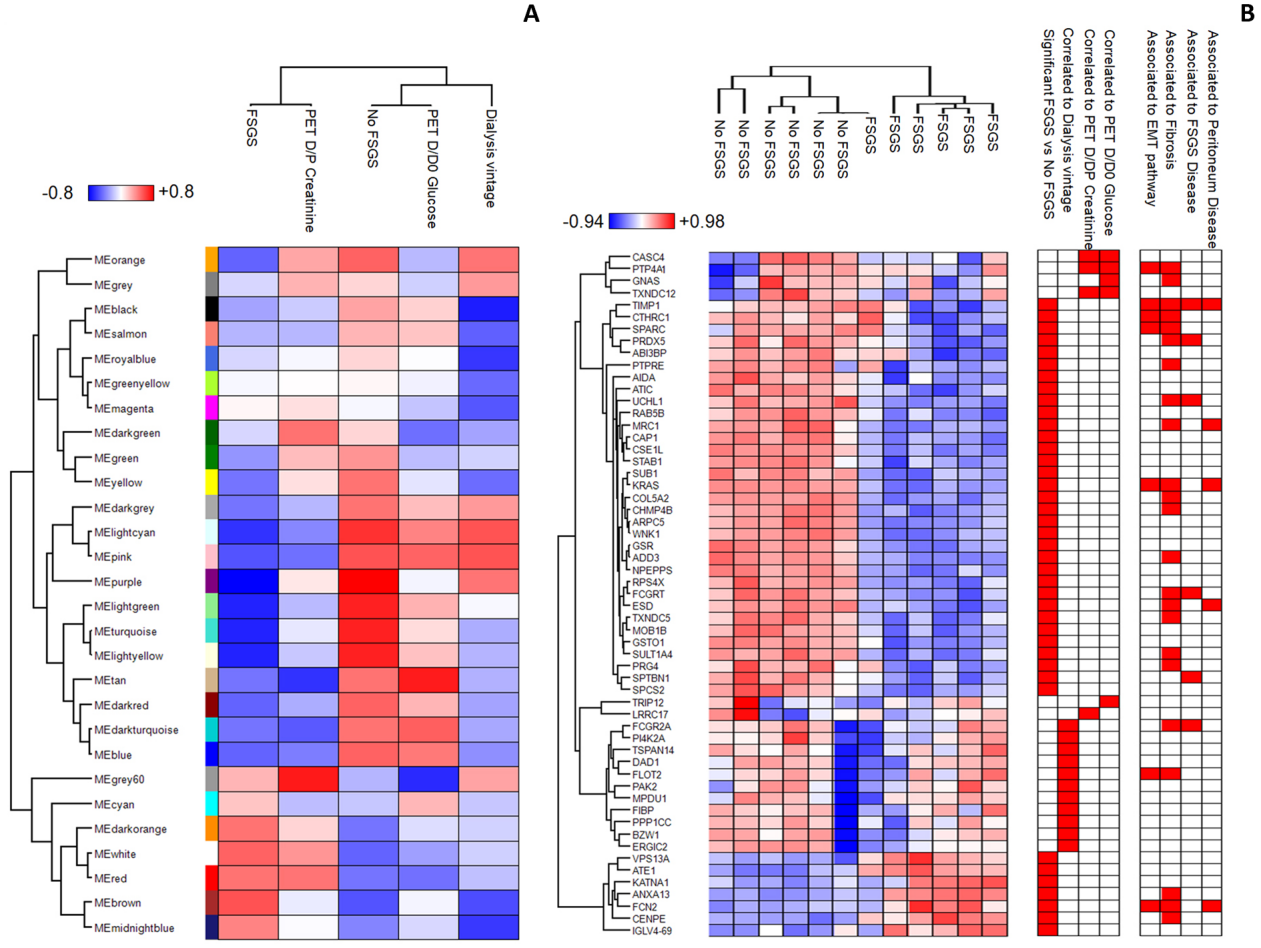
Weighted gene co-expression analysis of exosomes isolated from the peritoneal dialysis effluent of FSGS and No FSGS. (**A**) Heatmap of the correlation between module eigengenes and the clinical traits selected in the study. The grade of Spearman’s correlation coefficient ranged from − 0.8 (blue) to 0.8 (red). (**B**) Heatmap of 57 proteins in statistically significant correlation with at least one of the clinical traits selected in the study (Table 1). The correlation with each clinical trait is highlighted in red on the right of the heatmap.

This analysis revealed 28 modules encompassing proteins with similar co-expression profiles. To distinguish between modules, an arbitrary color was chosen for each module (Supplemental Table S1). The number of proteins included in each module ranged from 21 (white) to 273 (turquoise). The brown, purple, pink, tan and lightgrey modules showed closer relationships with the FSGS (r = 0.61), No FSGS (0.81), time of peritoneal dialysis (r = 0.61), PET D/D0 glucose (r = 0.81) and PET D/P creatinine (r = 0.81) respectively (Fig. 1A). No other statistical relationship was found with variables such as age, weight, height, body mass index and body surface area. The proteome profile of the 57 proteins significantly correlated (Spearman’s correlation coefficient values > 0.7 and two sides *p* values ≤ 0.05. See detail in Table 1) to either condition is visualized in a heatmap diagram (Fig. 1B). In the heatmap, each row represents a protein and each column corresponds to a sample. Normalized Z-scores of protein abundance are depicted by a pseudocolor scale, with red, white and blue indicating positive, absence and negative correlation, respectively. The tree dendrogram displays the results of unsupervised hierarchical clustering analysis, placing similar Spearman’s correlation coefficient values next to each other. Then, we applied the *T*-test to identify the proteins that best distinguish the type of disease.

**Table 1:** List of all highlighted proteins.

Protein IDs	Protein names	Gene names	Significant FSGS vs No FSGS	UP to 95% CI of FSGS vs No FSGS	AUC FSGS vs No FSGS	AUC P-value FSGS vs No FSGS	Fold Change FSGS vs No FSGS	*p *value FSGS vs No FSGS
P27216-2	Annexin A13	ANXA13	+	+	1	8.06	6.21 ± 0.01	8.06
Q15485	Ficolin-2	FCN2	+		1	3.75	2.5 ± 0.78	3.75
Q96RL7-4	Vacuolar protein sorting-associated protein 13A	VPS13A	+	+	1	3.34	4.87 ± 0.04	3.34
A0A075B6H9	Immunoglobulin lambda variable 4–69	IGLV4-69	+		1	3.45	2.14 ± 0.09	3.45
A0A087X0P0	Kinesin-like protein	CENPE	+	+	1	3.41	6.6 ± 0.06	3.41
Q5TA02	Glutathione S-transferase omega-1	GSTO1	+	+	1	5.2	− 4.35 ± 0.12	5.2
P61020	Ras-related protein Rab-5B	RAB5B	+		1	4.01	− 2.55 ± 0.08	4.01
P01116-2	GTPase KRas	KRAS	+	+	1	3.97	− 4.94 ± 0.83	3.97
D6RE83	Ubiquitin carboxyl-terminal hydrolase	UCHL1	+	+	1	3.87	− 3.8 ± 1.03	3.87
Q92954-5	Proteoglycan 4	PRG4	+		1	3.82	− 2.3 ± 0.24	3.82
Q9UEY8-2	Gamma-adducin	ADD3	+	+	1	3.68	− 6.17 ± 1.78	3.68
D6RCK3	MOB kinase activator 1A	MOB1B	+		1	3.66	− 2.95 ± 0.25	3.66
Q96CG8-3	Collagen triple helix repeat-containing protein 1	CTHRC1	+		1	3.65	− 2.16 ± 0.31	3.65
P30044-2	Peroxiredoxin-5, mitochondrial	PRDX5	+	+	1	3.59	− 3.75 ± 0.05	3.59
P31939-2	Bifunctional purine biosynthesis protein PURH	ATIC	+	+	1	3.42	− 3.11 ± 0.01	3.42
Q01082	Spectrin beta chain, non-erythrocytic 1	SPTBN1	+	+	1	3.3	− 3.45 ± 0.37	3.3
P55060-4	Exportin-2	CSE1L	+	+	0.97	3.77	− 6.32 ± 1.14	3.77
F5GY03	SPARC	SPARC	+	+	0.97	3.59	− 3.16 ± 0	3.59
P05997	Collagen alpha-2(V) chain	COL5A2	+	+	0.97	3.58	− 3.92 ± 0.79	3.58
P00390-2	Glutathione reductase, mitochondrial	GSR	+		0.97	3.44	− 2.87 ± 0.65	3.44
H7BZT7	S-formylglutathione hydrolase	ESD	+		0.97	3.26	− 2.6 ± 0.31	3.26
P55899	IgG receptor FcRn large subunit p51	FCGRT	+	+	0.97	3.23	− 3.43 ± 0.48	3.23
Q01518	Adenylyl cyclase-associated protein 1	CAP1	+	+	0.97	3.19	− 3.39 ± 0.8	3.19
A0A0A6YYL2	Sulfotransferase	SULT1A4	+		0.97	3.17	− 2.1 ± 0.19	3.17
Q96BJ3	Axin interactor, dorsalization-associated protein	AIDA	+		0.97	3.12	− 2.43 ± 0.14	3.12
E9PLK3	Puromycin-sensitive aminopeptidase	NPEPPS	+	+	0.97	3.09	− 3.43 ± 0.59	3.09
O15511	Actin-related protein 2/3 complex subunit 5	ARPC5	+	+	0.97	3.03	− 4.75 ± 1.26	3.03
Q5H9A7	Metalloproteinase inhibitor 1	TIMP1	+	+	0.97	3	− 3.88 ± 0.96	3
Q9NY15	Stabilin-1	STAB1	+	+	0.97	2.96	− 3.68 ± 0.35	2.96
P22897	Macrophage mannose receptor 1	MRC1	+	+	0.97	2.95	− 5.25 ± 1.52	2.95
P62701	40S ribosomal protein S4, X isoform	RPS4X	+	+	0.97	2.94	− 4.19 ± 0.67	2.94
A0A087WUC6	Signal peptidase complex subunit 2	SPCS2	+	+	0.97	2.89	− 3.73 ± 0.75	2.89
H0Y5C2	Arginyl-tRNA–protein transferase 1	ATE1	+	+	0.94	3.04	3.79 ± 0.82	3.04
O75449	Katanin p60 ATPase-containing subunit A1	KATNA1	+	+	0.94	3.39	5.17 ± 1.53	3.39
F5GWT4	Serine/threonine-protein kinase WNK1	WNK1	+	+	0.94	3.27	− 4.31 ± 0.78	3.27
Q8NBS9-2	Thioredoxin domain-containing protein 5	TXNDC5	+	+	0.94	2.92	− 4.15 ± 0.29	2.92
P53999	Activated RNA polymerase II transcriptional coactivator p15	SUB1	+	+	0.91	2.94	− 4.61 ± 0.79	2.94
Q9H444	Charged multivesicular body protein 4b	CHMP4B	+	+	0.91	2.92	− 3.44 ± 0.77	2.92
D3YTG3	Target of Nesh-SH3	ABI3BP	+	+	0.91	2.9	− 4.68 ± 1.6	2.9
P23469-3	Receptor-type tyrosine-protein phosphatase epsilon	PTPRE	+		0.91	2.9	− 2.1 ± 0.26	2.9
A0A087WU02	Endoplasmic reticulum-Golgi intermediate compartment protein 2	ERGIC2			0.71	0.16	− 0.47 ± 1.74	0.16
J3QLD9	Flotillin-2	FLOT2			0.66	0.41	1.14 ± 0.31	0.41
E9PAM4	Phosphatidylinositol 4-kinase type 2-alpha	PI4K2A			0.57	0.14		
F5GXX5	Dolichyl-diphosphooligosaccharide–protein glycosyltransferase subunit DAD1	DAD1			0.54	0.2	0.81 ± 0.84	0.2
F8VYE8	Serine/threonine-protein phosphatase	PPP1CC			0.6	0.07	− 0.14 ± 0.32	0.07
J3QS48	Mannose-P-dolichol utilization defect 1 protein	MPDU1			0.63	0.63	0.79 ± 1.13	0.63
O43427-2	Acidic fibroblast growth factor intracellular-binding protein	FIBP			0.54	0.14		
P12318-2	Low affinity immunoglobulin gamma Fc region receptor II-a	FCGR2A			0.51	0.18	0.6 ± 1.09	0.18
Q13177	Serine/threonine-protein kinase PAK 2	PAK2			0.69	0.59		
Q7L1Q6-2	Basic leucine zipper and W2 domain-containing protein 1	BZW1			0.66	0.04	− 0.04 ± 0.3	0.04
Q8NG11	Tetraspanin-14	TSPAN14			0.6	0.52	1.21 ± 0.85	0.52
A0A3B3ISR8	Protein tyrosine phosphatase type IVA 1	PTP4A1			0.51	0.04	0.12 ± 0.73	0.04
O95881	Thioredoxin domain-containing protein 12	TXNDC12			0.57	0.25		
Q6P4E1-2	Protein CASC4	CASC4			0.66	0.49		
Q8N6Y2	Leucine-rich repeat-containing protein 17	LRRC17			0.54	0.14		
P63092-3	Guanine nucleotide-binding protein G(s) subunit alpha isoforms short	GNAS			0.66	0.43		
Q14669-4	E3 ubiquitin-protein ligase TRIP12	TRIP12			0.69	0.16		

Protein IDs	Correlated to FSGS	Correlated to dialysis vintage	Correlated to PET D/P Cr	Correlated to PET D/D0 Glu	FSGS Sperman's coefficient	Dialysis vintage Sperman's coefficient	PET D/P Creatinine Sperman's coefficient	PET D/D0 Glucose Sperman's coefficient
P27216-2	+				0.98	− 0.24	0.26	− 0.31
Q15485	+				0.88	− 0.21	0.22	− 0.27
Q96RL7-4	+				0.85	− 0.31	0.18	− 0.2
A0A075B6H9	+				0.86	− 0.06	0.05	− 0.04
A0A087X0P0	+				0.86	− 0.32	0.26	− 0.32
Q5TA02	+				− 0.94	0.14	− 0.16	0.18
P61020	+				− 0.89	0.18	− 0.28	0.29
P01116-2	+				− 0.89	0.13	− 0.14	0.14
D6RE83	+				− 0.88	0.4	− 0.09	0.12
Q92954-5	+				− 0.88	0.03	− 0.02	0.05
Q9UEY8-2	+				− 0.87	0.14	− 0.1	0.12
D6RCK3	+				− 0.87	0.13	− 0.19	0.23
Q96CG8-3	+				− 0.87	0.3	− 0.12	0.21
P30044-2	+				− 0.87	0.26	− 0.23	0.24
P31939-2	+				− 0.86	0.08	− 0.28	0.3
Q01082	+				− 0.85	− 0.05	− 0.09	0.09
P55060-4	+				− 0.88	0.13	− 0.14	0.16
F5GY03	+				− 0.87	0.46	− 0.29	0.36
P05997	+				− 0.87	0.19	− 0.17	0.2
P00390-2	+				− 0.86	0.06	− 0.11	0.12
H7BZT7	+				− 0.84	0.31	− 0.3	0.34
P55899	+				− 0.84	0.08	− 0.27	0.28
Q01518	+				− 0.84	0.02	− 0.08	0.07
A0A0A6YYL2	+				− 0.84	0.14	− 0.33	0.33
Q96BJ3	+				− 0.83	0.12	0.05	− 0.01
E9PLK3	+				− 0.83	0.14	− 0.03	0.04
O15511	+				− 0.83	0.1	− 0.28	0.27
Q5H9A7	+				− 0.82	0.48	− 0.35	0.43
Q9NY15	+				− 0.82	− 0.09	− 0.18	0.18
P22897	+				− 0.82	− 0.08	− 0.17	0.15
P62701	+				− 0.82	0.17	− 0.16	0.19
A0A087WUC6	+				− 0.81	− 0.09	− 0.1	0.11
H0Y5C2	+				0.83	0	0.18	− 0.17
O75449	+				0.85	− 0.25	0.25	− 0.29
F5GWT4	+				− 0.84	0.13	− 0.22	0.23
Q8NBS9-2	+				− 0.82	0.01	− 0.33	0.33
P53999	+				− 0.82	0.1	− 0.15	0.14
Q9H444	+				− 0.82	0.16	− 0.28	0.28
D3YTG3	+				− 0.81	0.3	− 0.22	0.28
P23469-3	+				− 0.81	− 0.19	− 0.09	0.09
A0A087WU02		+			− 0.13	− 0.83	0	− 0.08
J3QLD9		+			0.27	− 0.86	− 0.11	0.01
E9PAM4		+			0.11	− 0.81	− 0.21	0.05
F5GXX5		+			0.15	− 0.88	0.03	− 0.13
F8VYE8		+			− 0.06	− 0.8	0.09	− 0.2
J3QS48		+			0.37	− 0.87	0.1	− 0.2
O43427-2		+			0.11	− 0.83	0.06	− 0.14
P12318-2		+			0.14	− 0.84	− 0.09	− 0.03
Q13177		+			0.36	− 0.86	− 0.15	0.03
Q7L1Q6-2		+			− 0.03	− 0.82	0.16	− 0.27
Q8NG11		+			0.32	− 0.81	0.01	− 0.13
A0A3B3ISR8		*	+	+	0.04	0.57	− 0.87	0.88
O95881			+	+	− 0.18	− 0.09	− 0.87	0.85
Q6P4E1-2			+	+	− 0.31	0.04	− 0.85	0.86
Q8N6Y2			+		− 0.11	0.07	0.8	− 0.76
P63092-3				+	− 0.28	0.15	− 0.74	0.82
Q14669-4				+	0.13	− 0.34	0.73	− 0.8

List of all proteins highlighted by means of Weight Gene Co-expression Network analysis, *T*-test, Partial Least Square discriminant analysis (PLS-DA) and Support Vector Machine (SVM). The symbol “+” identified the proteins highlighted in each analysis. Proteins Log_2_ Fold change and P-values are reported as mean ± standard deviation and − Log_10_ respectively. The order of proteins in the table correspond to the rank of priority in the discrimination of FSGS and No FSGS samples using SVM and PLS-DA (see detail in Supplemental Table S1).

*Pearman’s coefficient and *p *value respectively < 0.7 and < 0.05.

**Figure 2:**
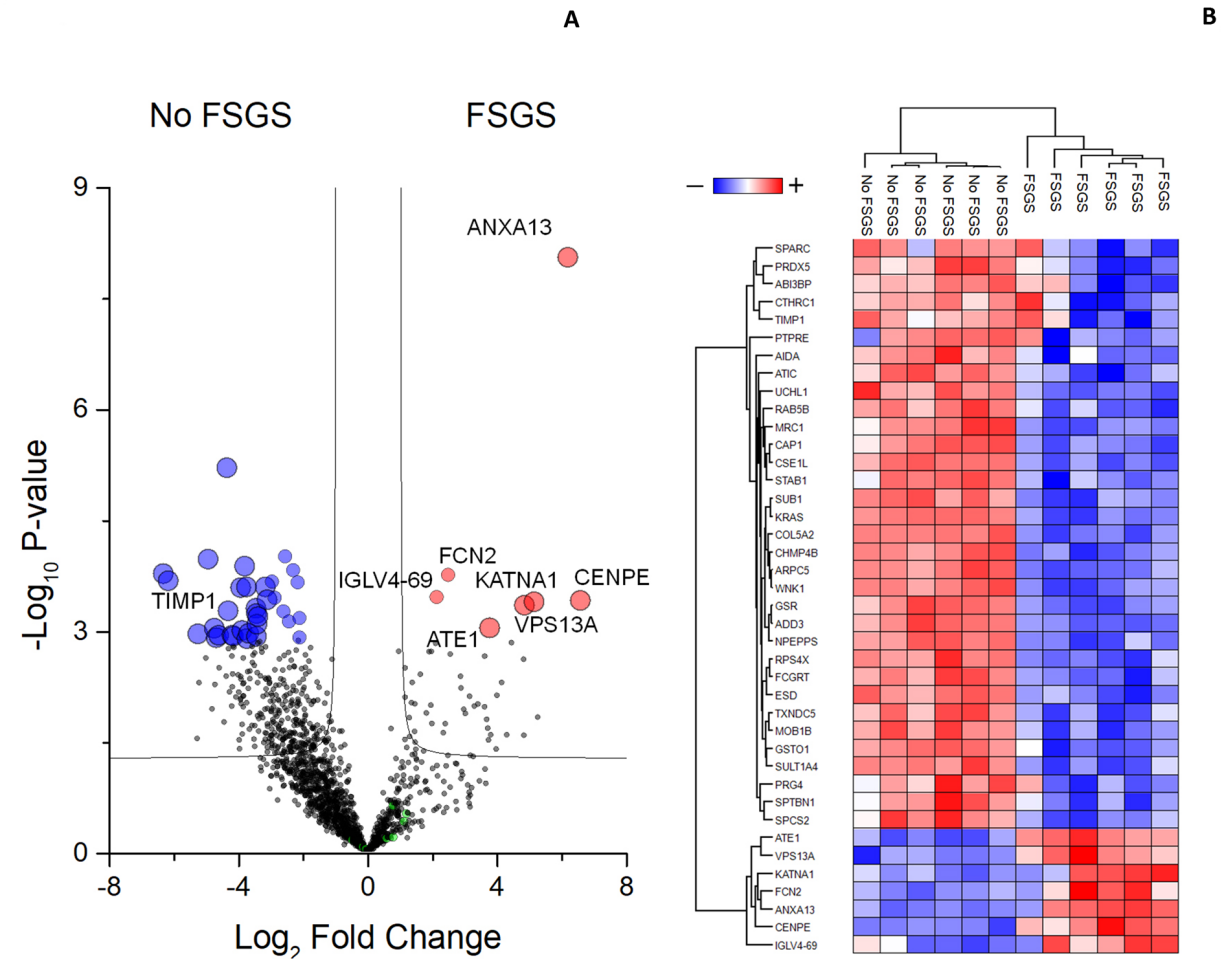
Volcano plot of univariate statistical analysis of peritoneal dialysis effluent exosomes from FSGS and No FSGS samples and heatmap of statistically significant proteins. (**A**) The plot is based on the fold change (log_2_) and their *p*-value (− log_10_) of all proteins identified in all samples. Red, blue, green and black circles indicate respectively the proteins with statistically significant up-regulation in FSGS or No FSGS samples, those associated with at least one of the clinical traits selected in the study and the non statistically significant. (**B**) Heatmap of 40 proteins statistically significant changed between FSGS or No FSGS samples (Table 1). Visual inspection of volcano plot, heatmap and their dendrograms demonstrates the ability of these proteins to distinguish between the FSGS and No FSGS samples.

A total of 40 proteins that maximized the discrimination between FSGS and No FSGS patients were highlighted (Fig. 2A and Table 1). Their expression profile after Z-score normalization is reported in a heatmap diagram (Fig. 2B). In the heatmap, each row represents a protein and each column corresponds to a sample. Normalized Z-scores of protein abundance are depicted by a pseudocolor scale with red, white and blue indicating positive, equal and negative expression, respectively. The tree dendrogram displays the results of the unsupervised hierarchical clustering analysis, placing similar sample/proteome profile values next to each other. SVM learning and PLS-DA were then used to make a rank list of these proteins (Table 1 and Supplemental Table S1). Among these, Annexin A13 (ANXA13) resulted the most promising up-regulated potential biomarker to distinguish peritoneal dialysis effluent exosomes of FSGS patients as it displays the highest *p *value in the *T*-test, maximal discrimination power in SVM and PLSDA, respectively with first position in rank and maximal value of variable importance (VIP) score. Instead, Tissue inhibitor matrix metalloproteinase 1 (TIMP1) was one of the most significant down-regulated protein in FSGS patients.

On the other hand, out of the 40 proteins maximizing the discrimination between the two groups of patients, 11, 5 and 4 proteins were highly correlated respectively with dialysis vintage, D/D0 glucose and with D/P creatinine at PET (Fig. 1B and Table 1). Among those related to D/D0 glucose and D/P creatinine values at PET, the expression of 4 proteins (PTP4A1, TXNDC12, GNAS and CASC4) was proportional to D/D0 glucose and inversely proportional to D/P creatinine, whereas 2 proteins (TRIP12 and LRRC17) had an opposite relationship (Table 1). In particular, protein tyrosine phosphatase type IVA 1 (PTP4A1) was the only one statistically related to dialysis vintage, D/D0 glucose and D/P creatinine at PET (Table 1).

The high diversity of protein profile expression between FSGS and No FSGS could imply a different biochemical role of these proteins. To assess this, we performed Gene Ontology (GO) enrichment analysis. This analysis identified 118 significantly enriched gene signatures. Among these, 61, 49 and 8 were enriched in FSGS, No FSGS or in both, respectively (Supplemental Table S2). These signatures are visualized using a scatter plot (Supplemental Figure S3). Interestingly, exosomes isolated from FSGS patients were enriched in proteins associated to their pathology (251 proteins^34^) and both groups were enriched in proteins already described in Exocarta (1873 proteins^35^), or associated to fibrosis (995 proteins^34^) and to EMT (251 proteins^34^) as shown in Supplemental Figure S3, S4.

### ELISA for ANXA13 validated proteomic results

**Figure 3:**
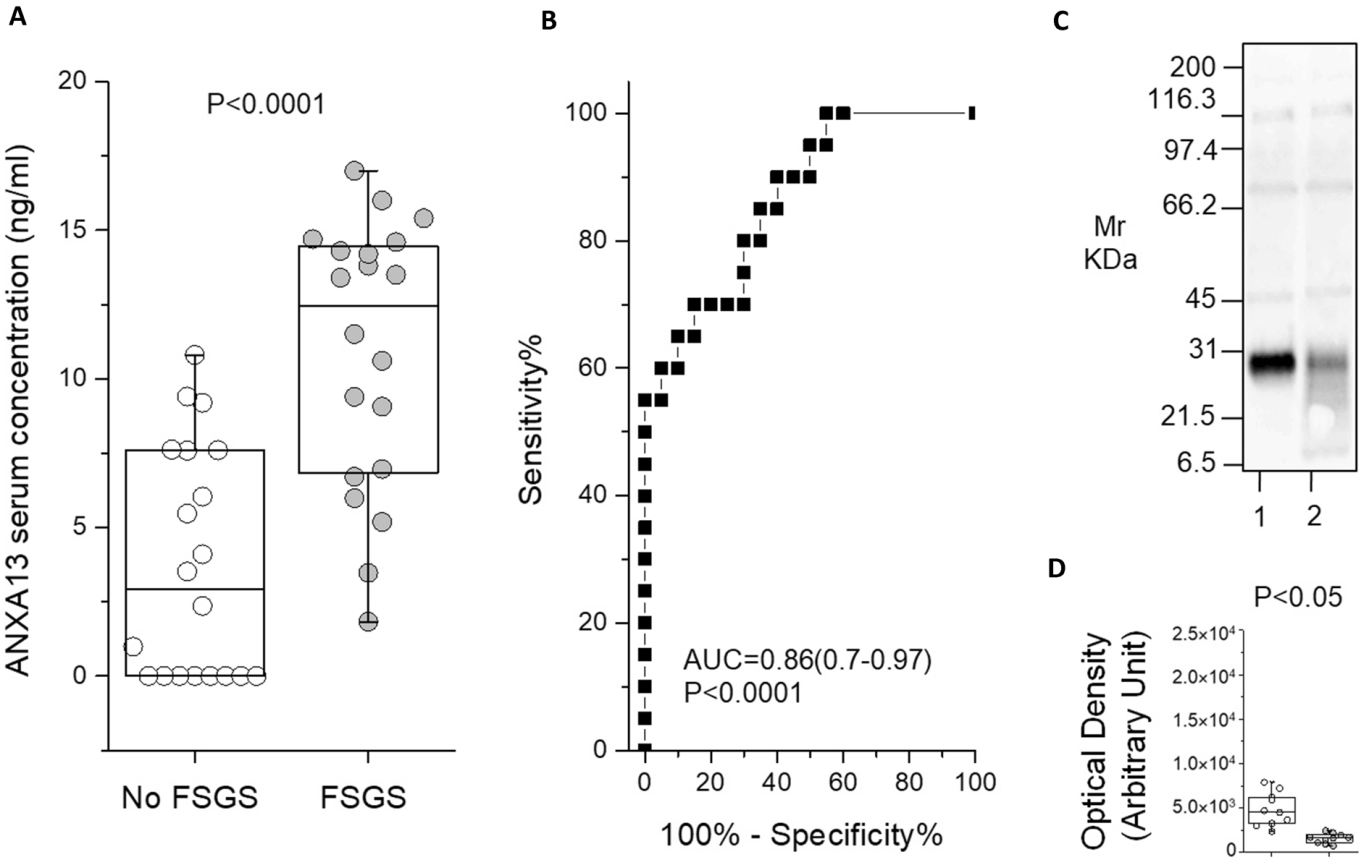
ANXA13 ELISA assay and TIMP1 western blot. (**A**) Box plot showing the median and interquartile range value of serum ANXA13 protein in an independent patients’ cohort. ANXA13 are more abundant in FSGS (grey circles) compared to No FSGS (white circles) patients (*p* < 0.0001); (**B**) ROC curve analysis for ANXA13 assay. (**C**) and (**D**) Representative western blot analysis of full length gel (8–16 T%) for TIMP1 in exosomes from with No FSGS (lane 1 or white circles) or FSGS (lane 2 or grey circles) and its densitometry analysis visualized as box plot (20 independent patients’ cohort). TIMP1 is more abundant in No FSGS compared to FSGS patients (*p* < 0.05).

Commercial ELISA Kits were used to determine the serum levels of ANXA13 in 20 FSGS versus 20 No FSGS patients. ANXA13 was statistically (*p* < 0.0001) more abundant in FSGS samples, as compared to No FSGS group (Fig. 3A). The median/IQR were respectively 12.4 (6.8–14.5) and 3 (0.1–7.9) ng/ml for FSGS and No FSGS. The cutoff, area under the curve (AUC), their confidence interval (CI) and *p *value of ROC curve were respectively 8.3 ng/ml, 0.86 (0.7–0.97) and *p* < 0.0001 (Fig. 3B). Finally, the resulting sensitivity (%), specificity (%) and likelihood ratio of the assay were respectively 70 (45.7–88.1), 85 (62.1–96.8) and 4.7.

### Western blot for TIMP1 validated proteomic results

Western blots was used to validate the proteomic results of TIMP1 expression in exosomes from PD effluent of FSGS and No FSGS patients. As shown in Fig. 3C TIMP1 was more abundant (*p* < 0.05) in exosomes from No FSGS patients compared to FSGS, namely 4.6E+03 (3.4E+03–6.1E+03) and 1.6E+03 (1.2E+0.3–1.9E+0.3) Optical Density Units, respectively (Fig. 3D). The AUC, its CI and *p *value resulted 0.89 (0.7–0.99) and *p* < 0.05, respectively. Finally, the precent sensitivity and specificity of the assay were 95 (69–98) (%), 60 (55–99) (%), respectively.

### PTP4A1 hyper-expression in TGF-beta-induced exosomes from PD effluent of patients with long dialysis vintage

**Figure 4:**
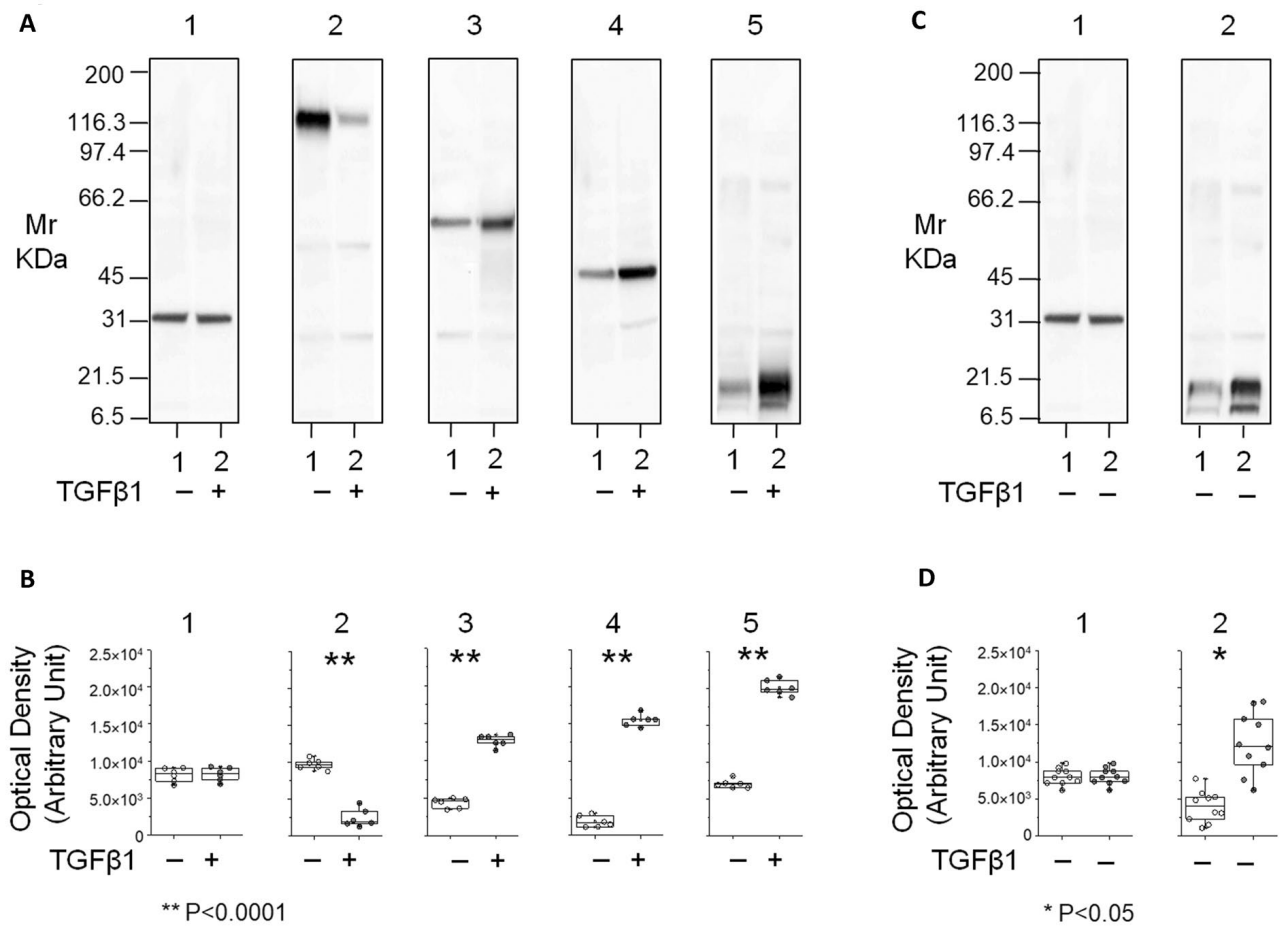
Analysis of the protein level of mesenchymal markers in human peritoneal mesothelial cells (HPMC) treated or not with TGFβ1 and in exosomes from peritoneal dialysis effluent of patients with short or long dialysis vintage. (**A**) Representative western blot analysis of full length gel (8–16 T%) for GAPDH (membrane and plot 1), E-cadherin (membrane and plot 2), Vimentin (membrane and plot 3), αSMA (membrane and plot 4) and PTP4A1 (membrane and plot 5) in whole lysate of HPMC treated with TGFβ1 (lane 2 or grey circle) or not (lane 1 or white circle) and (**B**) their densitometry analysis visualized as box plots (six biological replicates). HPMC treated with TGFβ1 showed a reduced expression of the epithelial marker E-cadherin and increased expression of VIME, α-SMA and PTP4A1 (*p* < 0.0001); (**C**) Representative western blot analysis of full length gel (8–16 T%) for GAPDH (membrane and plot 1) and PTP4A1 (membrane and plot 2) in exosomes from patients with short (lane 1 or white circle) or long (lane 2 or dark grey circle) dialysis vintage and (**D**) their densitometry analysis visualized as box plots (20 independent patients’ cohort). Exosomes with long dialysis vintage and high D/D0 glucose and low D/P creatinine values at PET or with short dialysis vintage and low D/D0 glucose and high D/P creatinine values at PET displayed the same PTP4A1 expression profile of HPMC treated or not with TGFβ1 (*p* < 0.05). GAPDH was used as loading control. All nitrocellulose membranes were cut perpendicular to the electrophoresis migration front to obtain a full length strips of samples and to allow the individually labeled and detection with the different antibodies.

To evaluate the potential role of PTP4A1 in fibrosis, immortalized HPMC were treated with TGFβ, a well-known inducer of fibrosis. The expression of PTP4A1 was up-regulated, together with reduced expression of the epithelial marker E-cadherin and increased expression of VIME and α-SMA, mesenchymal markers (Fig. 4A, B).

To confirm the correlation between PTP4A1 expression and dialysis vintage, its content was measured by western blot in PD exosomes from an independent group of 20 patients with same range of dialysis vintage of the patients used in the discovery approach. As shown in Fig. 4C PTP4A1 was statistically more abundant (*p* < 0.05) in exosomes from patients with long dialysis vintage and high D/D0 glucose and low D/P creatinine values at PET, compared to patients with short dialysis vintage but low D/D0 glucose and high D/P creatinine values at PET respectively being: 1.2E+04 (9.2E+03–1.6E+03) and 4.0E+03 (2.5E+0.3–5.2E+0.3) Optical Density Unit (Fig. 4D). The AUC, its CI and *p* value were 0.89 (0.7–0.98) and *p* < 0.05, respectively. Finally, the precent sensitivity and specificity of the assay were 95 (69–98) (%), 60 (55–99) (%), respectively.

## Discussion

Our proteomic study demonstrated, for the first time, that, despite the high overlap of the protein milieu between FSGS and No FSGS samples, the combined use of different analyses allowed a complete distinction (100% accuracy) of the proteomic profile of mesothelial exosomes in our two study groups.

Beyond the increasing evidence on the biological role of exosomes, the rationale of utilizing a purified fraction of PDE exosomes, is to reveal the huge amount of proteins probably masked by abundant high molecular weight proteins (*i.e.* Albumin). Indeed, a proteomic analysis on extracellular vesicles revealed a tenfold number of proteins^36^ compared to total^37^.

Out of the 2490 identified proteins, 10% (251) were FSGS-associated and 40% (995) were involved in fibrosis. Most of them were included in the transforming growth factor β (TGFβ) signalling pathway, playing a major role in EMT onset^16,38,39^. Additionally, the WGCNA algorithm identified a group of mesothelial exosome proteins that maximized the discrimination between FSGS and No FSGS and were highly correlated to peritoneal dialysis vintage, fibrosis, EMT and PM disease. Interestingly, metalloproteinase inhibitor 1 (TIMP1), down-regulated in FSGS, was significantly associated to all the above-mentioned conditions. TIMP1 is a metalloprotease (zinc metalloendopeptidase, MMP) inhibitor that binds the catalytic zinc ion, functioning in integrin signaling and in regulation of cell death and differentiation^40^. Matrix MMPs are linked to fibrosis, being the main groups of ECM-degrading enzymes^41^. In particular, MMP9 activates TGFβ1 and its expression correlates positively with experimental fibrosis^42^, suggesting its pro-fibrotic role^22^.

Other down-regulated proteins in FSGS were: CTHRC1 (Collagen triple helix repeat-containing protein 1), SPARC (secreted protein acidic and rich in cysteine), CHMP4B (fibrosisCharged multivesicular body protein 4b), COL5A2 (Collagen alpha-2(V) chain). CTHRC1 is a negative regulator of collagen matrix deposition and a promigratory protein involved in vascular remodeling, anti-fibrosis, and cancer, therefore its down-regulation is detrimental^21^. SPARC (secreted protein acidic and rich in cysteine) has been long known to possess anti-proliferative properties, acting against interstitial fibroblast proliferation by inducing ECM and predisposing to fibrosis^43,44^. CHMP4B is a component of the endosomal sorting complex involved in the formation of multivesicular bodies (MVBs) that generate exosomes as intraluminal vesicles by inward budding of their limiting membrane. COL5A2 is a regulatory fibrillar forming collagen and a key determinant in the assembly of ECM. COLV overexpression has been found in cancer, granulation tissue, inflammation, atherosclerosis, and fibrosis of lung, skin, kidney and liver.

By contrast, among up-regulated proteins in FSGS there were ANXA13 (Annexin A13), as most significant and promising potential biomarker, to distinguish peritoneal dialysis effluent exosomes of FSGS from No FSGS patients (with 100% accuracy), CENP-E (Centromere-associated protein E) and FNC2 (Ficolin-2).

Annexins comprise a family of proteins structurally characterized by the annexin repeat motif, able to bind to negatively charged phospholipids in a Ca^2+^-dependent manner. Annexins are classified into A–E groups, with group A expressed in vertebrates, comprising 12 members (ANXA1–A13, being ANXA12 unassigned). Annexins link cytosolic Ca^2+^ dynamics to cytoskeletal responses, being variably implicated in proliferation, differentiation, migration, therefore in pathologies as autoimmunity, and infection^45^. Here, ANXA 1,5,6,7,8,11 and 13 have been identified, with ANXA 1,5,6,7,8 down-regulated and 2,3,4,11,13 up-regulated. In particular, ANXA13 is a protein that can self-associate in a calcium-dependent manner and form complexes with proteins possessing EF hands motives. Annexin A13 is a myristoylated member of the family present in two splice variants (a and b expressed in polarized epithelial cells), well represented in the kidney. Annexin A13 is a lipid raft-associated protein that plays a role in membrane transport events and in the organization of membrane dynamics of epithelial cells^46^.

FNC2 is a member of the Ficolins, a member of the collectin family of proteins, able to recognize pathogen-associated molecular patterns (PAMPs) on microbial surfaces. Upon binding to their specific PAMP, ficolins may trigger activation of the immune system by either binding to cellular receptors for collectins or by initiating activation of complement via the lectin pathway. FCN2 could be involved in fibrosis by complement activation, associated to myofibroblast activation and fibrogenesis in various animal models of glomerulopathies and FSGS^47,48^. It has also been associated to the development of PD-induced arteriolar vasculopathy in children on chronic PD^49^.

CENP-E is a mitotic kinesin necessary for the mitotic microtubule capture at kinetochores, whose removal results in mitotic arrest.

Furthermore, mesothelial exosomes from FSGS patients expressed fewer proteins statistically associated with the length of dialysis treatment and/or PET test alteration. This appears in line with previous studies in which the protein milieu of extracellular vesicles obtained from PDE was analysed^25,36^. Notably, in one of these studies, the most enriched GO was “Exosome”^25^.

Data seem confirmed by the observation that HPMC treated with TGFβ1 showed increased expression of proteins associated with mesenchymal phenotype (VIME, α-SMA and PTP4A1) displaying the same PTP4A1 expression profile of the exosomes from patients with long dialysis vintage and high D/D0 glucose and low D/P creatinine values at PET. PTP4A1 belongs to a class of prenylated protein tyrosine phosphatases (PTP4A1/2/3) key promoters of TGFβ signaling in fibroblasts, involved in cell migration^50^. PTP4A1 was also shown to be able to activate EMT, in a model of intrahepatic cholangiocarcinoma (ICC)^50^. The under-expression of E-cadherin in face of overexpressed PTP4A1 is consistent with the report showing that PTP4A1 represses E-cadherin through the PI3K/AKT signaling pathway^51^. Although PTP4A1 has been already described in other fibrotic models, its role in kidney or peritoneal membrane disease has not been investigated until now.

Interestingly, PTP4A1 was related to both dialysis vintage and PET values. Notably, PTP4A1 is associated to high values of D/D0 glucose and low values of D/P creatinine, that identify the specific pattern of ultrafiltration failure type II, characterized by “ineffective transcapillary ultrafiltration associated with very low peritoneal transport rates”^52^. Pro-fibrotic mediators such as TGFβ1, and EMT seem to be involved in this process that leads to a diffuse hypopermeability of the PM. In rare and extreme cases, this type of ultrafiltration failure develops in EPS^53^.

Notably, the possibility to clearly discriminate PD patients with FSGS and those affected by other PRDs could be useful for the stratification of the 30% of children with ESKD who are referred for transplantation but had not undergone a renal biopsy, and around to 20% of which are diagnosed with possible glomerulonephritis^26,54^. In fact, notwithstanding the advances in immune-modulating and extracorporeal therapies, FSGS remains a challenge for pediatric nephrologists, as response to treatment is low and recurrence can occur after renal transplantation in a significant percentage of cases^13^.

Our data are consistent with the widely shared hypothesis that PDE is a promising source of protein biomarkers^55^. A seminal study demonstrated the presence of extracellular vesicles in PDE and examined them from 12 adult patients. The proteomic analysis of the extracellular vesicles showed an enrichment by size-exclusion chromatography of over 2,000 proteins normally masked by abundant proteins in PDE such as albumin, and identified about 3,700 proteins many of which involved in PM pathology, among which CD81, integrin 3A, ADAM10 (disintegrin and metalloproteinase domain-containing protein 10, that mediates the proteolytic cleavage of IL6R, releasing it) the exosomal marker protein ALIX (ALG-2-interacting protein 1), mesothelin and MUC16 (two markers for mesothelial cells), and CA-125^36^.

There are some limitations of this study that we have to underline. First of all, the small size of the studied patient population, and some differences in the clinical characteristics between the two groups, even if the impact of these differences didn’t affect the results of our cutting-edge analysis, based on SVM learning. The wide range of PD duration at the moment of the study, and the absence of evaluation of the impact of the treatment and renal function on our results.

In conclusion, the present proteomic study, although performed on a limited number of patients, demonstrated the existence of a multi-factorial biological machinery (mainly pro-fibrotic) in mesothelial cells of our pediatric patients affected by FSGS. However, this study represents a pilot, and the results, although supported by innovative and high-performance analysis methods deserve further prospective, proteomic and clinical studies to confirm the present data on a larger pediatric PD patient population, to clarify if patients with idiopathic FSGS may be more exposed to the risk of developing PM dysfunction and/or fibrosis when treated with long-term PD. Corroborate our findings, could be essential to eventuallyoptimize replacement therapy in this patient population, for example, by introducing the use of more biocompatible PD solutions known to reduce the activation of the pro-fibrotic machinery and mesothelial to mesenchymal transition or accelerating, as much as possible, their inclusion in the transplant waiting list for kidney transplantation to avoid peritoneal complications. Some of the identified proteins (such as PTP4A1) may be also proposed as biomarkers of mesothelial integrity.

## Materials and methods

### Patients and isolation of enriched exosome fractions

A total of 52 ESKD patients in PD treatment followed up at the Nephrology Department of the Gaslini Children’s Hospital were included in the study. Written informed parental consent was obtained before enrolment. The main demographic and clinical features are summarized in Table 2. The inclusion criteria were defined as follows: patients up to 18 years old, on stable PD for more than one month, without peritonitis in the three months preceding the study, and who had not received a previous kidney transplant. Twelve randomly selected patients were included in the proteomic analysis: six patients had primary focal segmental glomerulosclerosis as baseline nephropathy (FSGS group) and 6 have been affected by other diseases (No FSGS group) (Table 2). A group of 40 patients (20 FSGS and 20 No FSGS) were included in the validation part of the study.

**Table 2:** Clinical data of FSGS and No FSGS patients.

	FSGS	No FSGS	*p *value
Sex M/F	3/3	3/3	1
Age at test (year)	11.5 (9–15.75)	6.5 (1–13.75)	0.37
Weight (kg)	32.1 (25.68–50.65)	18.08 (9.89–35.78)	0.13
Height (cm)	144.5 (129.5–160.3)	107.4 (78.25–144.3)	0.18
Body surface area (m^2^)	1.1 (0.97–1.52)	0.7 (0.47–1.175)	0.09
Body mass index	16.6 (14.43–19.43)	15.7 (14.88–17.68)	0.87
Dialysis vintage (months)	9 (3.5–17.75)	11.5 (5.5–21.5)	0.82
Dwell volume (ml)	1200 (967.5–1475)	700 (350–1150)	0.12
PET D/P Creatinine	0.73 (0.7–0.88)	0.7 (0.51–0.94)	0.63
PET D/D0 Glucose	0.35 (0.22–0.37)	0.38 (0.1975–0.4875)	0.47
Glucose concentration PD solution (%)	1.74 (1.53–1.815)	1.36 (1.36–1.59)	0.06
Bicarbonate-lactate/lactate buffer (yes/no)	3/3	5/1	0.54
Hemoglobin (Hb) (g/dl)	9.4 (8.97–10.93)	11.45 (10.78–12.25)	0.03*
White blood cells (n°/cc)	5.24 (4.95–5.99)	6.105 (4.90–7.63)	0.39
Neutrophils (n°/cc)	2.72 (2.20–3.36)	3.03 (2.32–3.728)	0.7
Platelets (n°/cc)	236 (201.5–316.3)	336 (250.8–369.3)	0.24
Creatinine (mg/dl)	11.62 (10.47–14.77)	4.64 (2.5–8.33)	0.026*
Calcium (mg/dl)	9.13 (8.57–10.31)	10.29 (8.37–10.68)	0.24
Phosphates (mg/dl)	5.86 (5.52–7.23)	4.66 (3.83–5.27)	0.002*
Parathyroid hormone (PTH) (pg/ml)	159.5 (51.73–372.8)	194 (125–326)	0.66
Cholesterol (mg/dl)	172.5 (150.8–254.8)	235 (143–253.3)	1
Triglycerides (mg/dl)	147.5 (109.3–341.3)	168 (118.5–392.3)	0.82
Systolic blood pressure (mmHg)	130.5 (103–141.3)	102 (78–122.3)	0.09
Diastolic blood pressure (mmHg)	86.5 (73.5–96)	61 (56.2–81.7)	0.06
Treatment			
β-blocker (yes/no)	5/1	3/3	0.54
Angiotensin converting enzyme inhibitor (yes/no)	6/0	2/4	0.06
Calcineurin inhibitors (yes/no)	6/0	0/6	0.002*
Rituximab (yes/no)	4/2	1/4	0.24

Continuous variables are reported as median and (interquartile range). Statistically differences in continuous and discrete clinical variables between FSGS and No FSGS patients were determined respectively using Mann–Whitney test or Fisher’s exact test with a 2 × 2 contingency table. Two sides *p *values ≤ 0.05 were considered as significant.

PM function was evaluated through a 4-h peritoneal equilibration test (PET) conducted by using 1000 ml per m^2^ of patient’s body surface area of a 2.27% glucose PD solution^56^. Urea and creatinine dialysate-to-plasma concentration ratio (D/P) and glucose dialysate to initial dialysate concentration ratio (D/D0) were calculated.

Patients have been treated with automated peritoneal dialysis (APD) using biocompatible glucose-based solutions, with different glucose concentrations (1.36%, 2.27% and 3.86%) according to the required fluid removal, and with bicarbonate/lactate buffer.

The study was carried out in accordance to Italian and international ethical guidelines and approved by the Comitato Etico Regione Liguria (number: 408REG2014).

Sample collection was standardized by performing the analyses on the PDE obtained at the end of a 4-h PET (see above). Exosomes from mesothelial peritoneal cells were isolated by centrifugation plus immuno-magnetic beads affinity capture. Aliquots (100 ml) of PDE at PET were centrifuged at 22,000×*g* for 120 min at 16 °C to remove cells, debris, microvesicles and organelles such as mitochondria. Supernatants were then centrifuged at 100,000×*g* for 120 min at 16 °C to pellet the exosomes. The exosomal pellet was resuspended in 1 ml 0.25 M sucrose, loaded onto 1 ml 30% sucrose cushion and centrifuged at 100,000×*g* for 120 min at 16 °C. The pellet was rinsed in PBS and centrifuged again at 100,000×*g* for 120 min at 16 °C.The final pellet was stored at − 80 °C until use.

### Immuno-magnetic beads capture

The method is based on the capture of a specific subset of exosomes from peritoneal dialysis effluent using a biotinylated antibody and streptavidin magnetic beads.

Enriched exosomes fractions were mixed with polyclonal biotin-conjugated anti-human mesothelin (MSLN) antibody (LifeSpan BioSciences, Seattle, WA, USA) and incubated 4 h at RT with gentle rotation. Then, streptavidin Dynabeads (ThermoFisher) were added according to the procedure of the manufacturer. Briefly, exosome-antibody-dynabeads complexes were incubated for 30 min at RT with gentle rotation, placed on the magnet and rinsed five times to remove unspecific exosomes and unbound antibodies.Then, sample was removed from the magnet and bound exosomes collected by adding 250 µl of elution buffer. Finally, supernatant was centrifuged at 100,000×*g* for 120 min at 16 °C to pellet the exosomes pelleted. Such rinse/centrifugation cycle was carried out five times to obtain a clean anti-human mesothelin-positive exosome fraction. Size and purity of the isolated exosomes were assessed by DLS.

### Dynamic light scattering

Exosome size was determined by DLS using a Zetasizer nano ZS90 particle sizer at a 90° fixed angle (Malvern Instruments, Worcestershire, UK). The particle diameter was calculated using the Stokes–Einstein equation. For particle sizing in solution, exosome aliquots were diluted in 10% PBS and analyzed at a constant 25 °C.

### Western blotting

Expression of exosomal or human peritoneal mesothelial cells (HPMC) markers were detected by western blot. Aliquots of exosome fractions or whole lysate of HPMC were solubilized in 2% w/v SDS, 10% glycerol and 62.5 mM Tris–HCl pH 6.8 and separated by sodium dodecyl sulfate polyacrylamide gel electrophoresis (SDS-PAGE) and then transferred to a nitrocellulose membrane. Full length membrane was blocked, rinsed and cut perpendicular to the electrophoresis migration front to obtain a full length strips of whole samples and to allow the individually labeled and detection with one of the following primary human antibodies diluted in 3% (w/v) bovine serum albumin (BSA) in PBS containing 0.05% v/v Tween-20 (PBS-T): monoclonal anti-CD63 (Novus Biological, Littleton, CA, USA, 1:1000 clone H5C6), monoclonal anti-CD81 (Novus Biological, 1:1000 clone 1D6), monoclonal anti-CD45 (LifeSpan BioSciences, Seattle, WA, USA, 1:1000 clone 3G4), monoclonal anti-CD4 (Abcam. 1:1000 clone EPR19514), monoclonal anti-CD8 (Abcam, 1:1000, clone BLR044F), monoclonal anti-CD3 (Abcan, 1:1000, clone SP7), monoclonal anti-CD68 (Abcam, 1:1000, clone KP1), monoclonal anti-CD79a (Abcam, 1:1000, clone EPR3619), anti-mesothelin biotin-conjugated (LifeSpan BioSciences, Seattle, WA, USA, 1:1000), anti-E-cadherin (Santa Cruz Biotechnologies, CA, USA, 1:1000), anti-*α*-SMA (kindly provided by Professor G Gabbiani, 1:750), anti-vimentin (Novocastra, 1:1000), anti-PTP4A1 (ThermoFisher Scientific, 1:1000), anti-TIMP1 (Abcam, 1:1000) and anti-GAPDH (Sigma-Aldrich, 1:1000). After rinsing in PBS-T, the membrane was incubated with HRP-conjugated secondary antibodies (diluted 1:10,000 in 1% w/v BSA in PBS-T). Chemiluminescence signal was acquired and quantified using respectively the ChemiDoc and Quantity One software (Bio-Rad, Hercules, CA, USA). Gel electrophoresis was digitized by GS-800 Densitometer (Bio-Rad, Hercules, CA, USA).

### Mass spectrometry (MS) analysis

Samples were lysed, reduced and alkylated in 50 ul of iST-LYSE buffer (PreOmics) for 10 min at 95 °C and then digested over night at 37 °C with 0.7 ug Trypsin and 0.3 ugLysC. Digested samples were processed by iST protocol^57^.

Elution of the digested samples was performed with a 200 cm uPAC C18 column (PharmaFluidics) maintained at 40 °C in the thermostatic column compartment of an Ultimate 3000 RSLC. The peptides were separated with increasing organic solvent at a flow rate of 350 nl/min using a non-linear gradient of 5–45% solution B (80% CAN and 20% H_2_O, 5% DMSO, 0.1% FA) in 155 min.

MS data were acquired on an Orbitrap Fusion Tribrid mass spectrometer (ThermoScientific). MS1 was performed with Orbitrap detection at a resolving power of 120 K, while MS2 was performed with Ion Trap detection with Rapid Ion Trap Scan Rate. Top speed mode with a 2 s. cycle time was performed for data dependent MS/MS analysis, during which precursors detected within the range of m/z 375 − 1500 were selected for activation in order of abundance. Quadrupole isolation with a 1.6 m/z isolation window was used, and dynamic exclusion was enabled for 30 s. Automatic gain control targets was set at 4 × 10^5^ for MS1 and at 1 × 10^4^ for MS2 with 50 and 45 ms maximum injection times respectively. The signal intensity threshold for MS2 was 1 × 10^4^. HCD was performed using 28% normalized collision energy. One microscan was used for both MS1 and MS2 events.

Raw data were processed with MaxQuant^58^ software version 1.6.10.0. A false discovery rate (FDR) of 0.01 was set for the identification of proteins, peptides and PSM (peptide-spectrum match). For peptide identification a minimum length of 6 amino acids was required. Andromeda engine, incorporated into MaxQuant software, was used to search MS/MS spectra against Uniprot human database (release UP000005640_9606 April 2019). In the processing the Acetyl (Protein N-Term), Oxidation (M) and Deamidation (NQ) were selected as variable modifications and the fixed modification was Carbamidomethyl (C).

Whole Mass spectrometry data are friendly available at ProteomeXchange Consortium^59^ via the PRIDE58 partner repository with the dataset identifier PXD024556 (Reviewer account details: Username: reviewer_pxd024556@ebi.ac.uk, Password: 1BfsyHkc).

### ELISA assay

To quantify ANXA13 in undiluted serum of an independent group of 20 patients with FSGS and 20 No FSGS patients, commercial ELISA kit was used (Abnova, KA6081). Assay was performed following the manufacturer’s instructions. The Reference standards were run in triplicate and test samples were run in duplicate. Box plot was used to visualize the protein concentration. In box plot each point indicates the mean obtained from duplicate measurement. The lower detection limit was determined as the lowest protein concentration that could be differentiated from blank.

### Cell culture

Human peritoneal mesothelial cells (HPMC) were purchased from Creative Bioarray (Shirley, New York, USA). They were grown in DMEM/F12 medium supplemented with 10% FBS, penicillin (100 U/ml), and streptomycin (100 μg/ml), and maintained at 37 °C in a humidified incubator supplied with 5% CO_2_. After cells reached 70% confluence, they were cultured in serum-free medium in the presence or absence of 10 ng/ml TGFβ1 (R&D Systems, Minneapolis, MN) to 96 h and then processed for western blots. Six biological replicates have been done.

### Statistical analysis

After normalization, whole mass spectrometry data were analyzed by unsupervised hierarchical clustering using multidimensional scaling (MDS) with k-means and Spearman’s correlation, in order to identify outliers and dissimilarity between samples. Then, the normalized whole dataset was used to construct a co-expression network using the weight gene co-expression network analysis (WGCNA) package in R^20^. A weighted adjacency matrix was constructed using the power function. After choosing the appropriate β parameter of power (with the value of independence scale set to 0.8) the adjacency matrix was transformed into a topological overlap matrix (TOM), which measures the network connectivity of all proteins.

To classify proteins that display co-expression profiles into protein modules, hierarchical clustering analysis was conducted according to the TOM dissimilarity, with a minimum size of 20 proteins per module. To identify the relationship between each module and each clinical trait, we used module eigengenes (MEs) and calculated the Spearman’s correlation between MEs and the clinical traits, namely: dialysis vintage; PET values; D/D0 glucose and D/P creatinine; FSGS and No FSGS patients. A heatmap was then used to visualize each degree of relationship. Same analysis was done for each protein. Proteins were considered in correlation with at least one clinical traits with a significant (two sides *p *values ≤ 0.05 after Benjamini–Hochberg correction for multiple interactions) Spearman’s correlation coefficient values > 0.7.

To identify the hub proteins of modules that maximize the discrimination between FSGS and No FSGS samples, we applied *T*-test, machine learning methods such as non-linear support vector machine (SVM) learning, and partial least squares discriminant analysis (PLS-DA). For the *T*-test, proteins were considered to be significantly differentially expressed between two conditions with power of 80% and an adjusted *p *value ≤ 0.05 after correction for multiple interactions (Benjamini-Hochberg) and a fold change of ≥ 2. In addition, the proteins needed to show at least 70% identity in the samples in one of two conditions and area under the curve (AUC) in the received operating characteristic (ROC) analysis > 0.8. Volcano plots were used to visualize the expression fold change differences between FSGS and No FSGS samples.

In SVM learning, a fourfold cross-validation approach was applied to estimate the prediction and classification accuracy. Besides, the whole matrix was randomly divided into two parts: one for learning (65%) and the other (35%) to determine the prediction accuracy.

Finally, gene set enrichment analysis^60^ was done to build a functional proteins network based on their Gene Ontology (GO) annotations extracted from the Gene Ontology Consortium (http://www.geneontology.org/). The protein profile expression data were loaded in the dataset and a ranked list was assigned to each GO annotation/pathway. These ranks take into account the number of proteins associated with each gene signature with respect to all proteins, their mean of fold change and the *p* value after False Discovery Rate (FDR) correction for multiple interactions. These ranks are confined between − 1 and 1, corresponding to minimal and maximal enrichment in each group. In the two-dimensional scatter plot utilized to visualize this analysis, the points located on the straight line passing through the coordinates (1_x_, 1_y_) and (− 1_x_, − 1_y_) represent the equally enriched signatures. The distance from this line is proportional to the increase in signature enrichment in one of the two groups (over or under the straight line are the GO annotation/pathway positively enriched in FSGS or No FSGS samples respectively).

For the ELISA assay, Mann–Whitney U-test for unpaired samples was used to assess the difference in the concentration of the potential biomarker of FSGS samples. Results were expressed as medians and interquartile range (IQr). Receiver operating characteristic (ROC) curves were generated to assess the diagnostic efficiency of assay. AUC value was classified as: 0.5, not discriminant; 0.5–0.6, fail; 0.6–0.7, poor; 0.7–0.8, fair; 0.8–0.9, good and 0.9–1, excellent. Youden's index and Likelihood ratio were used to identify the cutoff and the diagnostic performance of each assay, respectively. Two sides *p *values ≤ 0.05 were considered as significant. All statistical tests were performed using Origin Lab V9 and the latest version of software package R available at the time of the experiments^61^.

## Supplementary Information

Supplementary Information 1.

Supplementary Information 2.
