# Supplementary material for: Proteomic profile of mesothelial exosomes isolated from peritoneal dialysis effluent of children with focal segmental glomerulosclerosis
Source: Sci Rep. 2021 Oct 21;11:20807. doi: 10.1038/s41598-021-00324-4 (PMC8531449; doi:10.1038/s41598-021-00324-4)
Supplement: Supplementary file 1 — Supplementary Information 1. [file 41598_2021_324_MOESM1_ESM.docx]

**Supplementary Files.**

**PROTEOMIC PROFILE OF MESOTHELIAL EXOSOMES ISOLATED FROM PERITONEAL DIALYSIS EFFLUENT OF CHILDREN WITH FOCAL SEGMENTAL GLOMERULOSCLEROSIS.**

Maurizio Bruschi^1#^, Edoardo La Porta^2#^, Isabella Panfoli^3^, Giovanni Candiano^1^, Andrea Petretto^4^, Enrico Vidal^5^, Xhuliana Kajana^1^, Martina Bartolucci^4^, Simona Granata^6^, Gian Marco Ghiggeri^1,7^, Gianluigi Zaza^6^ and Enrico Verrina^2^.

^1^Laboratory of Molecular Nephrology, IRCCS Istituto Giannina Gaslini, Genoa, Italy

^2^Dialysis Unit, Department of Pediatric, IRCCS Istituto Giannina Gaslini, Genoa, Italy and Department of Internal Medicine (DiMi), University of Genoa, Genoa, Italy

^3^Dipartimento di Farmacia (DIFAR), Università di Genova, Genoa, Italy

^4^Core Facilities - Clinical Proteomics and Metabolomics, IRCCS Istituto Giannina Gaslini, Genoa, Italy

^5^Department of Medicine DAME-Division of Pediatrics, University of Udine, Udine, Italy

^6^Renal Unit, Department of Medicine, University Hospital of Verona, Verona, Italy

^7^UO of Nephrology, Dialysis and Transplantation, IRCCS Istituto Giannina Gaslini, Genoa, Italy

#These authors have equally contributed

***Corresponding Author:** Dr. Maurizio Bruschi, PhD

Laboratory of Molecular Nephrology

IRCCS Istituto Giannina Gaslini

Via Gerolamo Gaslini, 5

16147 Genoa

Italy

Phone: +3901056363524

Mail: mauriziobruschi@yahoo.it

**Supplemental Figure 1. Characterization of exosomes purified from peritoneal effluent of FSGS and No FSGS patients. A)** Plot of exosome size distribution, as evaluated by dynamic light scattering. The plot shows a Gaussian distribution profile with a mean peak at 100 ± 5 nm. No statistical differences were observed between the exosomes isolated from FSGS and No FSGS patients. **B)** Representative western blot analysis of full length gel of antigen profile of exosomes isolated from FSGS and No FSGS patients. Whole exosomes from FSGS (lanes 1) and No FSGS (lanes 2) patients were analyzed by detecting mesothelin (membrane 1), CD81 (membrane 2), CD63 (membrane 3),CD45 (membrane 4) CD3 (membrane 5), CD79a (membrane 6), CD68 (membrane 7), CD4 (membrane 8) and CD8 (membrane 0). Blue colloidal coomassie stained was used as loading control (gel 10). No difference in size and antigen profile were observed between exosomes isolated from FSGS or No FSGS patients. Nitro cellulose membrane were cut perpendicular to the electrophoresis migration front to obtain a full length strips of samples and to allow the individually labeled and detection with the different antibodies.

**
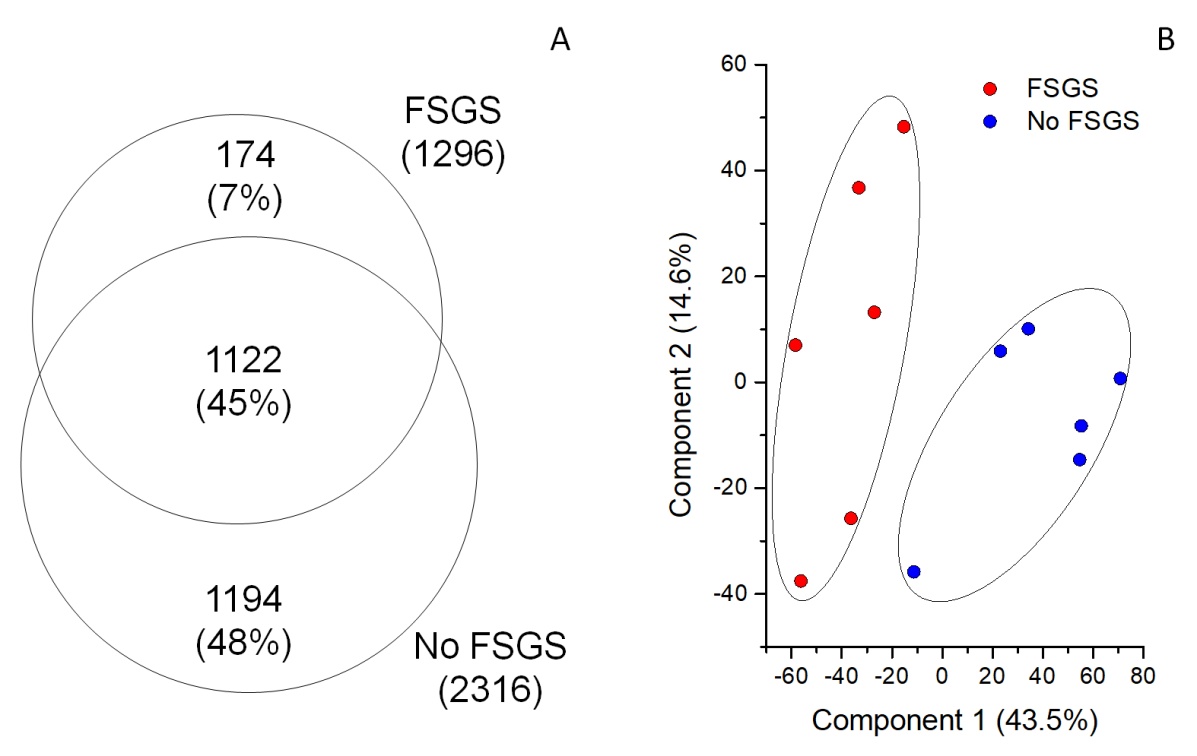
**

**Supplemental Figure 2. Venn diagram of total proteins identified and multidimensional scaling analysis of exosomes isolated from the peritoneal dialysis effluent of FSGS and No FSGS patients. A)** Venn diagram shows common and exclusive proteins identified in exosomes of FSGS and No FSGS patients. The numbers represent the distinct proteins in the overlapping and non-overlapping areas**. B)** Two-dimensional scatter plot of multidimensional scaling analysis. The plot shows the un-supervised cluster analysis of exosome (circle) of FSGS (red) and No FSGS (blue) samples using all identified proteins in all samples (circle). The ellipse shows the 95% of CI of two clusters. No outliers were detected.

**
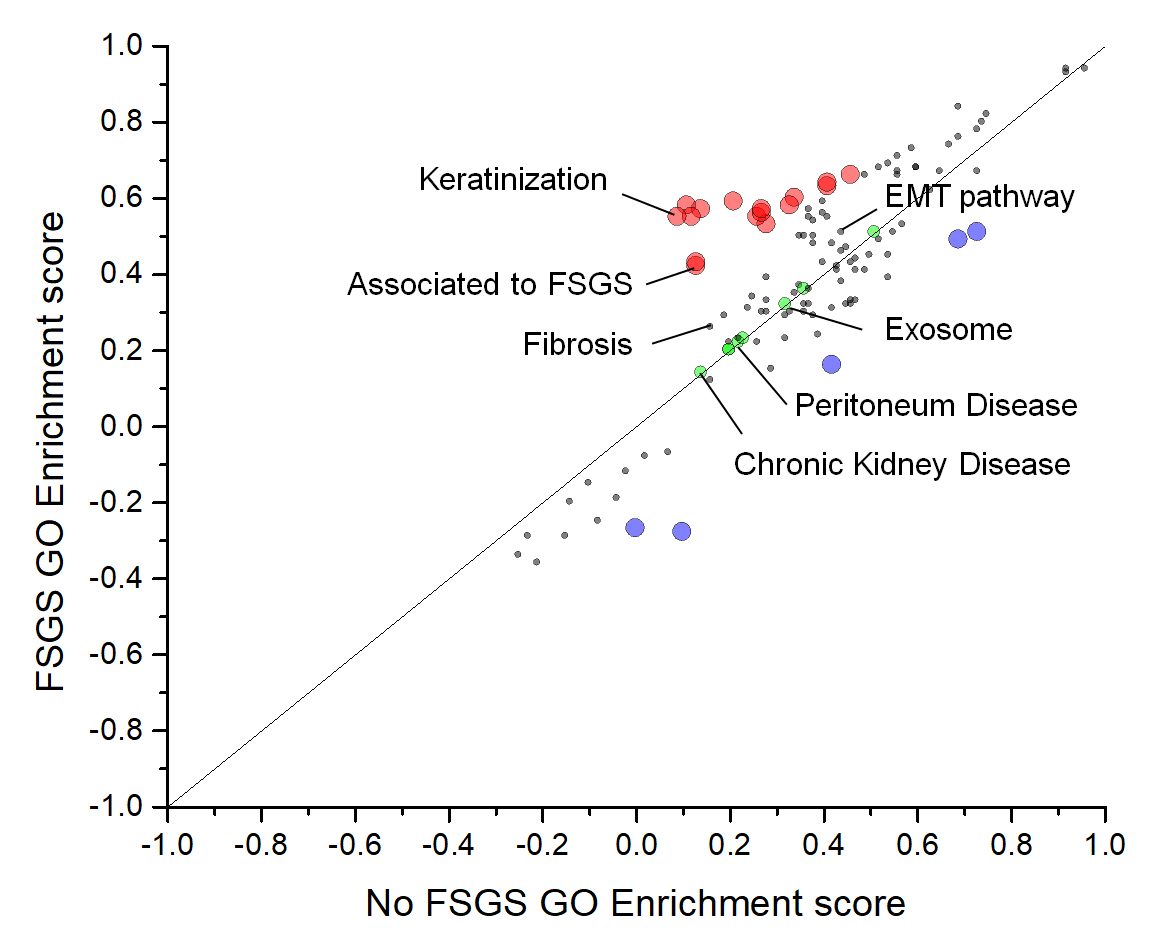
**

**Supplement Figure 3. Gene Ontology annotation enrichment analysis.** Plot shows the enriched signatures in exosomes isolated from peritoneal dialysis effluent of FSGS and No FSGS samples. In the graph, the points located on the straight line passing through the coordinates (1x,1y) and (-1x,-1y) are the equally enriched signatures, while those above or under this line are positively enriched in FSGS or No FSGS, respectively. Red and blue circles are the signatures enriched above the 95% of CI respectively in FSGS and No FSGS samples (see detail in Supplemental Table 1).

**
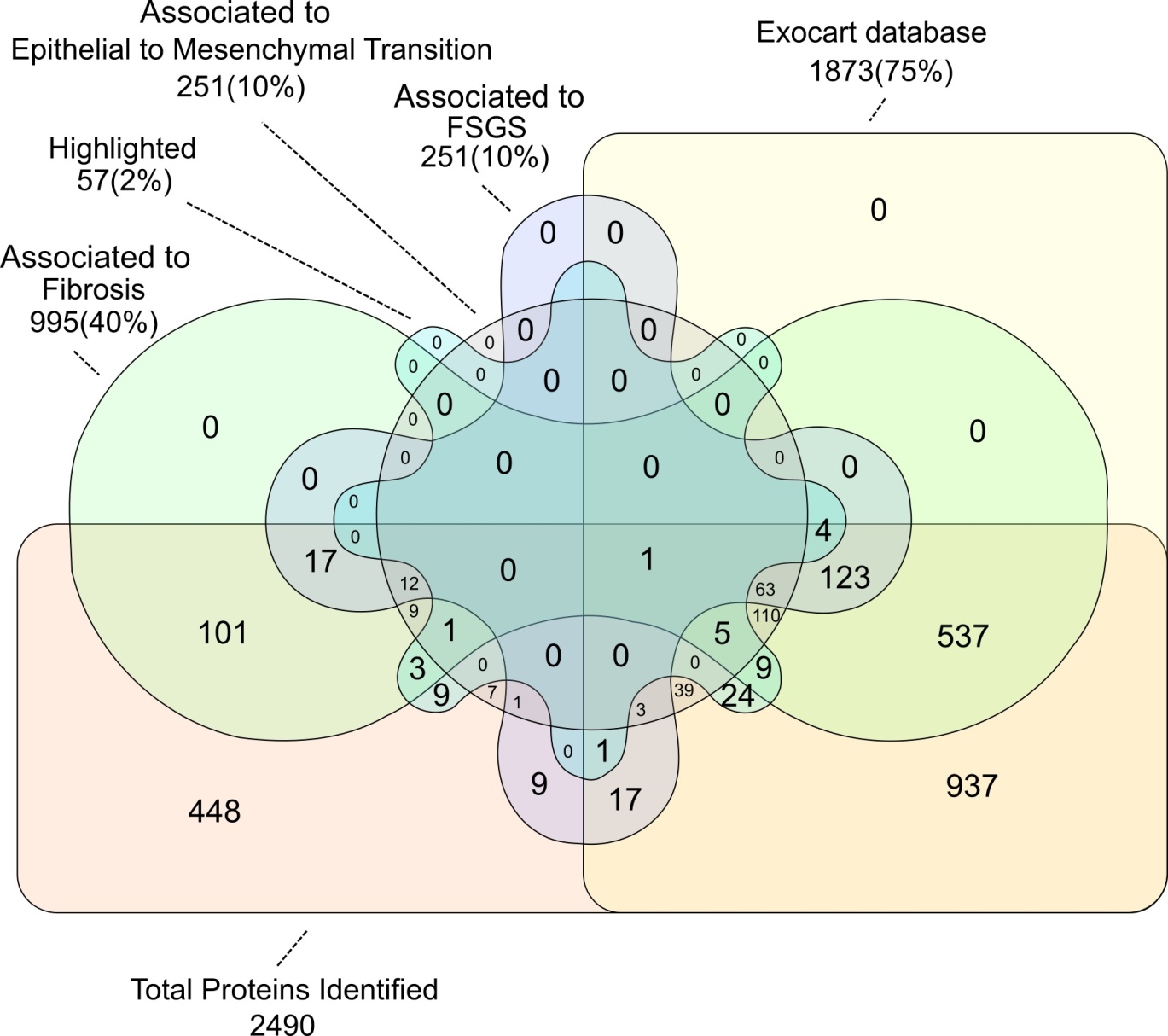
**

**Supplement Figure 4. Venn diagram of total proteins identified and the database resulted enriched in GO analysis.** Venn diagram shows common and exclusive proteins identified in all samples and the main database identified in the enrichment analysis. The numbers represent the distinct proteins in the overlapping and non-overlapping areas.
